# Ophthalmic medicolegal cases in Upper Egypt

**DOI:** 10.1186/1755-7682-2-1

**Published:** 2009-01-02

**Authors:** Ismail A Wasfy, Ehab IA Wasfy, Tarek A Aly, Alaa A Abd-Elsayed

**Affiliations:** 1 Ophthalmology Department, Assiut University Hospital, Assiut, Egypt; 2 Public Health and Biostatistics Department, Faculty of Medicine, Assiut University, Assiut, Egypt

## Abstract

**Purpose:**

To describe the pattern of ophthalmic medicolegal cases with emphasis on cases of assault, and to acquaint ophthalmologists with rules pertaining to expert testimony and medical reports.

**Methods:**

A retrospective study was carried out to review files of 247 medicolegal cases from Upper Egypt seen by the senior author in 8 years. These were classified categorically and were analyzed from various characteristics and aspects. The scheme for examination of subjects and for formulating the medicolegal report is described.

**Results:**

The different categories were assault in 224 cases (90.5%), military recruitment evasion in 8 cases (3.25%), occupational disability claims in 8 cases (3.25%) and medical malpractice in 7 cases (3%). Thirty two cases (13%) presented with alleged functional visual loss, of them 25 cases (10%) were malingering. Traumatic lens subluxation or dislocation was seen in 37 (13.5%) cases and phthisis and atrophia bulbi was the presenting sign in 55 (22.3%) cases. Twenty percent of assault cases were females. There were no differences in incidence between the provinces in Upper Egypt. Assault tools inflicted injuries are described, as well as the outcome of these cases. Claims against military recruits could not be substantiated. Occupational claims for damages were false. Alleged medical negligence cases were rejected based on accepted standards of care and not on unexpected complications.

**Conclusion:**

Medical reports have to be structured, detailed, accurate and unbiased. Data in this work are useful for statistical and planning purposes in the medicolegal domain.

## Introduction

In the Egyptian Judicial system, which adopts the continental model, the Ministry of Justice appoints medicolegal consultants to give opinion in litigations belonging to the criminal or civil law.

These consultants are considered expert witnesses and must be competent to give expert testimony which has three distinct requirements [1]:

1. The testimony must be composed of scientific, technical, or other specialized knowledge.

2. The testimony must assist the fact finder in understanding the evidence or resolving a factual dispute in the case.

3. The witness must be qualified to render the opinion which may be by knowledge, skill, experience, training or education.

The expert witness may be requested by the police, medicolegal authority or the court to examine victims of assault and other claimants and to give evidence in the form of a written statement (report or deposition) which is often accepted by the court as documentary evidence. A medical report has to be detailed, accurate, and unbiased [2].

Usually victims or claimants (plaintiffs) are seen several weeks or months after the assault or the cause for the litigation. In these cases it is imperative to ask for and to read carefully the initial medical report issued by the resident or casualty officer who first saw the case, as well as the other medical and hospital papers, x-ray films and other diagnostic images [3].

An expert witness may sometimes be summoned to appear in court to clarify points in the report, or for cross-examination by the defendant lawyer [3].

If the medicolegal consultant is summoned to court, a not very pleasant experience, he has to sustain orally that which he originally wrote [2]. In court the doctor should appear serious, authoritative, and wearing sober professional clothing. He has to speak in a clear, firm and loud enough voice. He must not be over-talkative, hostile, angry, rude or sarcastic during questioning [2].

## Materials and methods

The senior author worked as Forensic Consultant in Ophthalmology in Upper Egypt for 8 years. During this period he serially dealt with 247 medicolegal cases. This article is a retrospective study of the files of these cases kept in the private clinic of the senior author in Assiut.

Victims of assault and other claimants were sent by the prosecutor to the consultant's clinic accompanied by an official to be examined and a report issued. A litigation file accompanied the victim containing a preliminary report of the forensic clinician, the police report about the incident and all the relevant medical papers and hospital records.

The prosecutor indicated the aspects on which he wishes most information and the points to be clarified in the report. This usually included a description of the lesions, the tool inflicting the injury and whether the lesion conforms to the alleged cause of the incident, and whether a permanent infirmity was the outcome.

A systematic examination was carried out as any clinical case including personal, present, past and family history and functional examination of both eyes. A thorough systematic objective examination was performed on eyes, addenda, face and body in general.

The date, time and place of the examination were noted. All documents sent with the case were read carefully, copied and filed. The medicolegal report was issued on the same occasion of examining the case and given to the official who brought the case after obtaining his signature. The report may be issued within 1–3 days and sent to the authorities requesting it by registered post.

### The report as issued was formed of 3 sections

**Section 1: **began with the doctor's name, qualifications, affiliation, address, and date of the report. The name, sex, age and occupation of the person examined came next. The address was omitted for security reasons. The date, time and place of the examination were reported. The number of the litigation, district, year and legal classification (e.g. criminal, etc....) followed. A brief account of the reason for the examination was given and a short note of the circumstances of the incident as stated by the police or prosecutor was mentioned.

**Section 2: **included details of the claimant's complaint which led to the examination, present and past medical history, and any longstanding handicap or chronic illness. Then followed the results of the subjective and objective examination of the two eyes in detail. Positive as well as negative findings were stated, occasionally with the help of sketches. A note indicating that the face, skull and the remaining body surfaces were examined and the results of this examination were included.

**Section 3: **The opinion concluded the statement, and included an indication of the age of injuries, the conclusions to be drawn from these injuries and whether the findings were "consistent" with the complaint. A note of whether the condition has settled down completely or not was added. Permanent infirmities were indicated, assessed and computed. The last sheet of the report was signed as well as the bottom of each sheet if the report was composed of several sheets.

Case files were kept in order, and were later scrutinized regarding different aspects. These were tabulated by the authors, explained, and analyzed.

Data entry and analysis were done using the Statistical Package for the Social Sciences (SPSS), version 15 (SPSS Inc, Chicago, IL, USA).

## Results

247 medicolegal cases were serially seen in this study over a period of 8 years. The mean work load was 32 cases per year.

The cases came from 6 governorates as shown in table 1

**Table 1:** No. of cases related to the population of 6 governorates.

**Governorate**	**Cases**	**Population**	**Percentage**
**Assiut**	73	3,495,000	0.00209
**Sohag**	79	3,886,000	0.00203
**Qena**	66	2,994,000	0.00220
**Aswan**	24	1,141,000	0.00210
**New Valley**	4	173,000	0.00231
**Red Sea**	1	190.000	0.00053

Total	247	11,879,000	

Categorically the 247 medicolegal cases fell into 4 groups. The majority were cases of assault (no. 224) (90.6%). Military evasion cases were 8 (3.3%). Medical malpractice cases were 7 (2.8%) while industrial workers litigations were 8 (3.3%).

The youngest patient was 7 years old and the eldest was 84 years of age. 70.9% of cases were in the age range from 20–59 years.

Most of our patients were males 197 (79.8%). While females were only 50 (20.2%).

Regarding laterality, the right eye was affected in 112 (45.3%) and the left eye in 109 (44.1%) patients respectively, while both eyes were affected in 26 (10.5%) patients.

As for the alleged tool or agent for the injury, table 2 shows this in detail.

**Table 2:** Alleged tool or agent causing trauma

**Tool**	**Cases**	**(%)**	**Salient Cause**
**Blunt**	157	63.6%	More than 50% by stick (shuma)
**Sharp**	44	17.8%	36% by axe or knife
**Chemical**	9	3.6%	Hydrosulphuric acid in one case
**Firearm**	14	5.7%	Mostly gunshot
**Blast**	2	0.8%	Explosion
**Malpractice**	7	2.8%	Medical or pharmaceutical
**Occupational hazard**	9	3.6%	Variable

**Total**	247		

The places were the assault happened are depicted in table 3

**Table 3:** Place of accident

**Place**	**No.**	**(%)**
**Farm**	13	45.7
**Street**	90	36.43
**House**	22	9.0
**School**	11	4.64
**Work**	5	2.2
**Hospital**	6	2.4

**Total**	247	100

Trauma at school occurred in 11 cases (10 school children and one school teacher). The causative tool was a stick (wooden) in 3, dividers in 2, pen in 4 cases and a plastic triangle in 2 cases. The teacher was assaulted by a colleague using a stick.

The injury affected the eye only in 110 (44.5%) patients, while eye and face were affected in 99 (40.1%) patients, 9 (3.6%) patients had eye, face and body injuries and 29 (11.7%) patients had eye and body injuries.

Alleged blindness was the presentation in 32 (12.9%) patients, of them 25 (10.1%) patients were malingerers and we could not confirm if the case was malingering or pathological in 7 (2.8%) cases.

More than half of our cases 138 (55.9%) presented to us between 3 and 6 month from the time of the assault while only 21 cases (8.5%) presented in the 1^st ^3 months and 67 (27.1%) cases presented to us between 6 and 12 months from the time of assault. Late presenters after one year were 32 (13%) patients.

- Lid injury causing scars and/or cosmetic blemish and in 8 cases canalicular obstruction occurred in 61 cases (24.7%).

- Blow-out fracture of the orbit occurred in one case.

- Homonymous hemianopia occurred in one case.

- Globe injury occurred in 184 cases (60%).

- Open globe cases were 63 (25.5%) caused either by a rupture globe or a globe laceration, and ending in enucleation in 8 cases (3.2%) and atrophia bulbi and/or phthisis in 55 cases (22.3%).

More than one closed globe lesion was commonly present in the same globe accounting for a discrepancy between the number of lesions (217) and the number of globes (121) as shown in table 4.

**Table 4:** Closed globe lesions in 121 eyes

**Lesion**	**Numbers**
**Corneal scar**	68
**Traumatic cataract**	46
**Subluxation dislocation**	37
**Aphakia**	19
**After cataract**	6
**Traumatic glaucoma**	7
**Retinal detachment**	23
**Choroidal rupture**	5
**Macular hole**	2
**Optic atrophy**	4

**Total**	217

Visual acuity (aided) was < 3/60 in the right eye in 109 cases (44%) and in the left eye in 116 cases (47%). These patients are considered blind and have a 35% visual handicap.

## Discussion

A doctor may become involved in legal actions as an "expert witness" to provide information or opinion to assist the legal process. Some clinicians will have more interest in this type of work than others, motivated by considering this work as interesting and professionally challenging and that it is a duty to colleagues, to the court and to the running of the legal system [4].

The cons of this work are that the time spent in medicolegal work reduced the ophthalmologist's ability to improve his professional chances.

Remuneration for expert opinion is not comparable with time spent developing a busy private practice within ophthalmology [5]. Also serious consequences await the expert witness making a knowingly false statement [3].

In this work, 247 medicolegal cases were serially examined in 8 years.

The number of cases from each governorate related to the population was almost identical as all belongs to Upper Egypt. It would be enlightening to know comparative figures from the Delta.

The majority of patients were males, as females accounted for 20% of cases. This is most probably due to social habits keeping women sheltered in houses and also to the culture of giving women due respect and condemning a man assaulting a woman.

The tools inflecting the injury were multiple (no. 60). The majority were blunt tools of which the stick (Shuma) takes precedence (valid % 31.2). This may be due to its importance in Upper Egyptian folklore as it gives its holder dignity and security. It is his weapon in violence, and joy in combat game and festival dance.

Few truly comparable studies of the prevalence and incidence of ocular medicolegal trauma cases were published.

Prof. Attiah [6] gave a figure of 19.4% for the stick as tool in assault cases.

Sharp tools were implicated in 18% of trauma cases in this work whereas the figure was 2% in Prof. Attiah work [6]. This may be due to chronological difference leading to changing attitudes in the population.

The places were the assault happened is shown in table 3. Ten school children were injured in school, may be due to lack of supervision during play. Prevention of physical punishment by teachers adds safety.

In work, house, school and sports preventive measures reduce the incidence of trauma cases [7].

55.4% of our cases were injured in their eye and other parts of their body e.g. face skull or body.

The statement should include a description of all injuries whether in the eye, adnexa or other parts of the body with sketches for the eye, face and body if possible [3].

More than half of our cases 138 (55.9%) presented between 3 and 6 months from the time of assault while only 21 cases (8.5%) presented in the first three months, and 67 (27.1%) cases presented between 6 and 12 months from the time of assault. Late presenters after one year were 32 (23%) patients.

As features of the original injuries usually change with time due to healing, repair and remodeling, it is imperative to securitize the initial medical report written by a casualty officer so as to have a mental picture of the original injury. In this connection it is imperative for the junior doctors to document trauma cases properly in their medical reports [8].

Medicolegal consultants should not be fooled by victims of assault. Feigning may take several forms. Malingering of visual loss (bilateral or unilateral) is common and may be discovered by the various tests for malingering [9].

Other patterns of malingering are exaggeration of visual defect as happens in assaults causing corneal opacities. These may be discovered by the disproportion between the visual acuity claimed and the physical signs.

Another pattern of malingering is replacement of an old accident or disease with a new assault result. Scrutiny of the same or other eye may reveal the truth.

Fortunately claims of medical negligence were quite few (no. 7, 2.8%) in this study.

In litigations for alleged medical negligence it is the onus of the plaintiff to prove establishing the doctor-patient relationship, breach of the applicable standard of care, he must connect the negligent act or omission with the damage suffered [10]. For negligence to be proved, the professional behavior of the doctor must have fallen short of the minimum which the patient in entitled to expect from a doctor of that particular experience, in those particular circumstances [10].

The possibility of involvement with a medical liability problem threatens all physicians. Familiarity with the claims encountered by others my enable ophthalmologists to avoid similar claims [11,12]. Luckily this possibility is less in Egypt than in the USA or European countries. Also the possibility is much less in ophthalmic practice than in other specialties (e.g. obstetrics and gynecology) [13].

Eight cases were seen with alleged evasion of military recruitment by causing corneal opacities in one eye. However in all these cases it was impossible to substantiate the allegation of the military authority. Courts are in general agreement that expert testimony stating that a conclusion is "possible" does not meet the standard for admissibility with respect to the party who bears the burden of proof. A doctor's testimony that a certain thing is "possible" is no evidence at all [1].

In this work serious consequences occurred in cases of assault. 63 (25.5%) cases ended with loss of the eye with enucleation or phthisis. 217 closed eye lesions were recorded, the majority leading to impaired vision.

Early and proper treatment of trauma cases and advancement in techniques, equipment, instruments and sutures help in reducing the toll of the aforementioned grave consequences.

For evaluation of visual disability several factors should be taken in consideration, i.e. corrected V.A. in the two eyes, the visual field, limitation of ocular movements, loss of accommodation and the cosmetic appearance [14].

## Conclusion

The medicolegal consultant is an "expert witness" to the court and should not be unduly partisan. Certainly the "whole truth" is to be sought and he should not agree to unethical editing of his report.

The medicolegal report forms the basis for the medical evidence in court. It has to be structured, detailed, accurate and unbiased. Wherever possible it should be typed on A_4 _or foolscap paper. The use of a word processor to check for errors is recommended.

Everything in the report must be absolutely true, as any false or even reckless statements may lead the doctor into disciplinary trouble or even a criminal prosecution.

The medical witness in court should "Dress up, stand up, speak up and shut up"!

Assault caused grave consequences in the eye. Phthisis and atrophy in 22.3% of cases and enucleation in 3.2% of cases. 217 closed globe lesions leading to impaired vision were encountered in this study.

Commonest tools inflicting injury are stick, stone, brick, fist, firearm, knife and axe in descending order.

## Competing interests

The authors declare that they have no competing interests.

## Authors' contributions

IAW carried out the patients' examination and reporting, EIW assisted in reporting the cases and the manuscript writing, TAA assisted in reporting the cases, AAA-E assisted in reporting the cases, general coordination, drafting of the manuscript, writing the final manuscript and provided important suggestions.

All authors read and approved the final manuscript.

## References

[B1] Piorkowski JD (2001). Legal Medicine Chapter 10 Medical testimony and the expert witness, American College of Legal Medicine Textbook Committee (eds).

[B2] Knight B, Knight B (1997). Simpson's Forensic Medicine Chapter 1 The Doctor and the Law.

[B3] Clarke M, Mc Lay WDS (1998). Clinical Forensic Medicine Chapter 5, The Doctor in Courted.

[B4] Taylor D (2003). Is medicolegal work a duty?. Br J Ophthalmol.

[B5] Leitch RJ (2003). Is medicolegal work a duty?. Br J Ophthalmol.

[B6] Attial MAEH (1949). The solimanic lecture about the medicolegal aspects of eye trauma. Bull Egyptian Ophthalmol Soc.

[B7] (2004). American Academy of Ophthalmology, Basic and Clinical Science Course, Chapter 11 Ocular trauma, epidemiology and prevention, section 13 International Ophthalmology, ACO staff (eds) San Francisco.

[B8] Abdel-Maaboud RM Some Studies in Medical Responsibility, MD Thesis, Recommendations, A prepared model of the primary traumatic report 1997 Faculty of Medicine.

[B9] Miller BW (1973). A review of practical tests for ocular malingering and hysteria. Surv Ophthalmol.

[B10] Hamm JW, Shelton P, Lee DA, Higginbotham EJ (1999). Clinical Guide to Comprehensive Ophthalmology, Chapter 17 Risk Management.

[B11] Bettman JW (1990). Seven hundred medicolegal cases in Ophthalmology. Ophthalmology.

[B12] Brick DC Risk Management Lessons from a review of 168 Cataract Surgery Claims. Surv Ophthalmol.

[B13] Bettman JW, Mackenzie Freeman H (1979). Ocular Trauma, Chapter 6 The medicolegal implications of the management of trauma.

[B14] Faye EE, Riordan-Eva P, Whitcher JP (2004). Vaughan & Asbury's General Ophthalmology, Appendix 1 Visual Standards.

